# Synthesis of Paeonol-Ozagrel Conjugate: Structure Characterization and *In Vivo* Anti-Ischemic Stroke potential

**DOI:** 10.3389/fphar.2020.608221

**Published:** 2021-02-01

**Authors:** Jing Zhang, Miaomiao Jiang, Hui Zhao, Lan Han, Yu Jin, Weidong Chen, Jianqing Wang, Ziyu Zhang, Can Peng

**Affiliations:** ^1^ College of Pharmacy, Anhui University of Chinese Medicine, Hefei, China; ^2^ Institute of Pharmaceutics, Anhui Academy of Chinese Medicine, Hefei, China; ^3^ Anhui Province Key Laboratory of Pharmaceutical Preparation Technology and Application, Hefei, China; ^4^ Department of Pharmacy, the Fourth Affiliated Hospital of Anhui Medical University, Hefei, China; ^5^ Chaohu Jinchen Pharmacy Co., Ltd., Shanghai Haihong Industrial Group, Chaohu, China

**Keywords:** ischemic stroke, paeonol-ozagrel conjugate, synthesis, partition coefficient, cytotoxicity, oxidative stress, inflammatory cytokines, chemical structure characterization

## Abstract

Ischemic stroke is a common neurological disease that can lead to mortality and disability. The current curative effect remains unsatisfactory because drug accumulation in the diseased areas is insufficient as a result of the unique blood–brain barrier. Therefore, much attention has been paid to develop a novel therapeutic compound, paeonol-ozagrel conjugate (POC), for ischemic stroke. Then, POC was successfully synthesized by conjugating of paeonol and ozagrel as mutual prodrug. A series of *in vitro* characterizations and evaluations, including high - resolution mass spectroscopy, nuclear magnetic resonance spectroscopy, partition coefficient, and assessment of cytotoxicity against PC12 cells, were performed. Pharmacokinetic study demonstrated POC is eliminated quickly (t_1/2_ = 53.46 ± 19.64 min), which supported a short dosing interval. The neurological score, infarct volume, histopathological changes, oxidative stress, inflammatory cytokines levels, and TXA_2_ levels also were evaluated *in vivo* in middle cerebral artery occlusion (MCAO) rats. All results showed that POC had a significant curative and therapeutic effect on ischemic stroke, as evaluated by the middle cerebral artery occlusion. Overall, POC can be expected to become a new drug candidate for the treatment of ischemic stroke.

## Introduction

Ischemic stroke is one of the most common and potentially lethal cerebrovascular disease in adults worldwide, with substantial morbidity and mortality (Sheng et al., 2015; Su et al., 2017). It has attracted more and more attention in the field of drug discovery (Yu et al., 2017). The unique blood–brain barrier (BBB) in the central nervous system (CNS) hinders the treatment of ischemic stroke because of the poor efficiency of drug delivery (Yue et al., 2014). Specifically, therapeutic outcomes remain poor as a result of insufficient drug accumulation in the lesion site. Therefore, great attention has been paid to develop novel anti-ischemic stroke agents that can enter the brain well (Zhang et al., 2017).

Ozagrel, a thromboxane A_2_ (TXA_2_) synthase inhibitor, is used in several countries to treat ischemic stroke (Ishitsuka et al., 2009; Park et al., 2015). TXA_2_ is a potent vasoconstrictor that promotes platelet aggregation (Imamura et al., 2003). The imidazole ring of ozagrel drives its selective inhibition (Celli et al., 2004). Ozagrel decreases TXA_2_ production and has antiplatelet and antithrombotic effects, which can inhibit cerebral thrombosis by inhibiting platelet aggregation during treatment of a cerebral infarction (Koumura et al., 2011; Xu et al., 2017).

Natural compounds are the most potential source of lead molecules for development as clinically utile drugs (Zhong et al., 2016). Recent scientific research places emphasis on the use of natural compounds of medicinal herbs to treat ischemic stroke in the field of drug discovery (Wu et al., 2010). Paeonol is an active ingredient of Moutan Cortex Radicis that offers various great bioactivities and few side effects (Hsieh et al., 2006). It is a visible improvement of cerebral infarction volume and neurological deficits owing to anti-inflammatory and antioxidative effects (Liao et al., 2016; Zhao et al., 2018).

A mutual prodrug is designed as conjugated or associated drugs from two or more drugs/pharmacophores which have specific pharmacological action (Tsogoeva, 2010; Redasani and Bari, 2012). Through a logical combination of already known drugs and synthetic methods to produce new drugs that exhibit the desired effect, it could lead to a unique strengthening of activity along with minimizing the side effects (Bansal and Silakari, 2014; Shaveta et al., 2016). In the development of promising drug candidates in the field of medicinal chemistry, hybridization of drugs with complementary pharmacological action represents one of the most effective ways to design new leading candidates (Pawelczyk et al., 2018).

**FIGURE 1:**
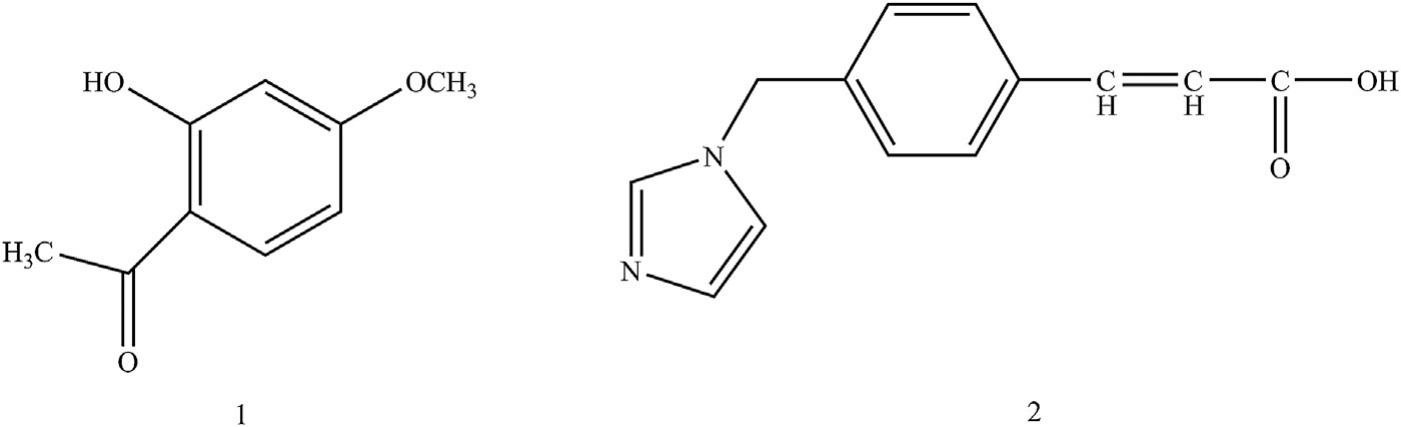
Structures of paeonol (1) and ozagrel (2).

In the present work, paeonol and ozagrel were conjugated by the concept of the mutual prodrug to produce a novel compound, which named POC. Structures of paeonol and ozagrel were shown as Figure 1. In addition, we established a series of indicators to evaluate whether POC has curative effect on ischemic stroke evaluated by the middle cerebral artery occlusion (MCAO) rat model *in vivo*.

## Materials and Methods

### Chemical Synthesis

Ozagrel (6.84 g) was dissolved in 110 ml of dichloromethane (CH_2_Cl_2_) and dropped with 0.5 ml of dimethylformamide (DMF). A mixture solution of thionyl chloride (15 ml) and CH_2_Cl_2_ (30 ml) was added dropwise with stirring at room temperature, and the mixture was dropped over about half an hour. The mixture was stirred for 2.5 h at 77°C. The degree of chlorination of ozagrel was determined on a thin layer of adsorbent material. At the end of the chlorinated, the reaction mixture was evaporated to remove excess SOCl_2_ and CH_2_Cl_2_ in a rotating evaporator. Then, 20 ml of CH_2_Cl_2_ was added to the residue and suction filtered.

**FIGURE 2:**
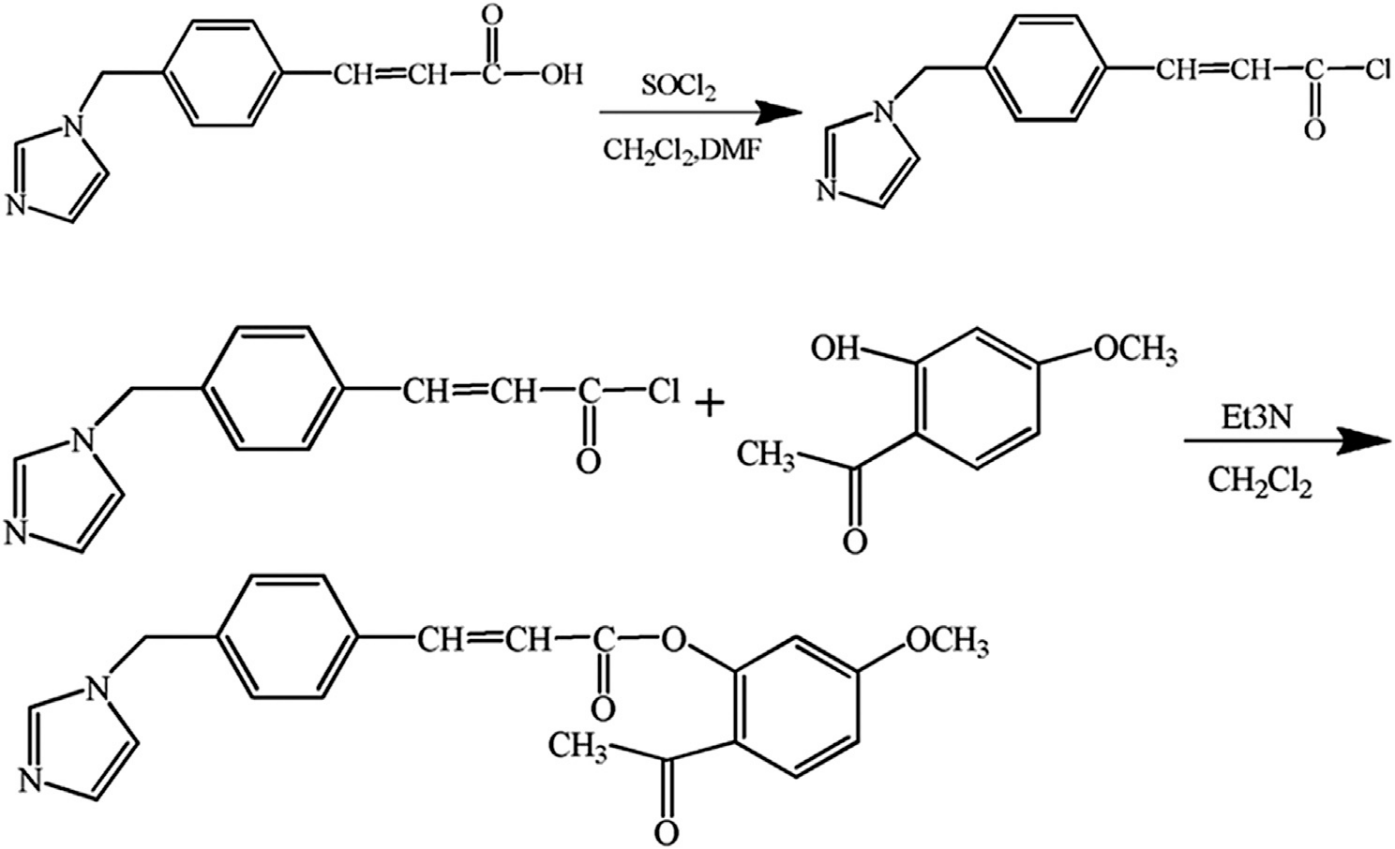
Structures and synthesis of POC. Reagents and conditions: ozagrel, paeonol, DMF, thionyl chloride, CH_2_Cl_2_, and trimethylamine.

Paeonol (4.99 g) was dissolved in 60 ml of CH_2_Cl_2_ and 10.5 ml of trimethylamine. Then, chlorinated ozagrel (mixed in 40 ml CH_2_Cl_2_) was added slowly with stirring and an ice bath. The mixture was agitated at room temperature for 2.5 h. At the end of the reaction, 90 ml of water was added to remove water–soluble impurities. Then the aqueous layer was extracted three times with a volume of dichloromethane, and the obtained dichloromethane was dried with anhydrous sodium sulfate. The eluate was concentrated by vacuum evaporation in a rotary evaporator under reduced pressure at a pressure 0.1 MPa and a temperature of 35°C. The concentrated solution was separated and purified through a chromatography column with ethyl acetate as the elution. Structures and synthesis of POC were shown as Figure 2. The effluent was monitored at 252 nm by UV-spectrophotometer. The high–molecular–weight fraction was lyophilized to provide the product of POC as a pale white powder.

### Characterization and HPLC Determination of POC

The structure of POC was characterized and confirmed by high - resolution mass spectroscopy (HR - MS) and nuclear magnetic resonance (NMR) spectroscopy. High - performance liquid chromatography (HPLC) was used to determine the purity of POC (WondaCract ODS C_18_, 46 mm × 150 mm, 5 μm; eluent, methanol:H_2_O = 70:30).

### Determination of Partition Coefficient

The partition coefficient of POC was established by the shake-flask method between n–octanol and an aqueous phase (Redasani and Bari, 2012). The aqueous phase buffered with phosphate (pH 7.4, 100 ml) and n–octanol (100 ml) was mixed well, placed in a shaking air bath for 72 h, and then left to equilibrate until the two saturated phases completely separated. POC (5 mg) was dissolved in 50 ml of saturated n-octanol and diluted with the saturated aqueous equally. The mixture was placed in an oscillator and shaken for 1 h until the two phases separated. Concentration was analyzed by HPLC method and then the partition coefficient was calculated as the ratio of the concentration of POC in n–octanol to aqueous. All experiments were made in triplicate.

### Cell Viability

Rat PC12 pheochromocytoma cells were obtained from the Cell Resource Center, Peking Union Medical College. PC12 cells were grown as monolayers in tissue culture flasks at 37°C under a humidified, 5% CO_2_ atmosphere in the high–glucose Dulbecco’s Modified Eagle Medium (DMEM) medium.

The CCK8 (MedChemExpress, United States) method was used to estimate the cytotoxic effects of POC. Before treatment, the cells were seeded onto 96-well culture plates at an appropriate density according to the experimental protocol. After 24 h, the medium was replaced with fresh medium containing varying concentrations (37.5, 75.0, 150.0, 300.0, 600.0, 1,200.0 μM) from the POC serial dilutions. And PC12 was allowed to grow for another 24 h. After incubation, the cell viability was evaluated using the CCK8 assay. The absorbance was read at 450 nm on a microplate reader. These cell viability experiments were made in triplicate.

### MCAO Model and Drug Administration

Male Sprague Dawley (SD) rats weighing among 220 g–230 g (Anhui Medicine University, Hefei, China; certificate number: SCXK20190,003) were housed under *ad libitum* access to food and water with a standardized light-dark cycle condition (25 ± 2°C, 12–hour light/dark cycle). All the experimental protocols were performed in accordance with the Institutional Animal Care and Use Committee of Anhui University of Chinese Medicine (Animal ethics number: AHUCM–rats–2019001). We used the minimal number of study animals (n = 6 rats in each group for different experiments).

The rats were fastened upon the operating table in a supine position after being anesthetized with 2% pelltobarbitalum natricum (50 mg/kg). After disinfection of the skin within close proximity of the operative region, the right common carotid artery (CCA), external carotid artery (ECA) and internal carotid artery (ICA) were exposed with blunt dissection. The CCA was temporarily ligated and the ECA were permanently ligated (Liu et al., 2016). A nylon monofilament surgical thread was introduced into the ICA to block the MCA when the blunted distal end met resistance. After 2 h of occlusion, reperfusion was accomplished to restore blood supply to the MCA area by removing the filament (Li et al., 2009; Zhai et al., 2017).

Ozagrel sodium (OS) is used clinically more often than ozagrel because of its improved solubility, so it was used rather than ozagrel for these experiments. Rats were split randomly into five groups, including the sham, model, paeonol, OS and POC group. After 2 h of reperfusion, the paeonol and OS groups received the drug by intravenous injection at a single dose of 3 mg/kg (Imamura et al., 2003; Park et al., 2010; Gai et al., 2019), which the doses of paeonol and OS uesd previously were benificial in rats and the dose of OS is within the clinical dose range. Rats in the POC group were injected intravenously in the tail vein with a dose of 4.5 mg/kg according to a calculation of the molar mass ratio to OS. An equal volume of 0.9% saline was administered in sham and model groups.

### Pharmacokinetic Study

Six rats were given POC (4.5 mg/kg) by intravenous injection, then, an approximately 1.0 ml sample of blood was obtained from the fossa orbitalis vein of rats and placed into heparin–rinsed EP pipes according to a programmed schedule at 3, 5, 10, 20, 30, 45, 60, 120, and 240 min after dosing.

Plasma (80 μL) was mixed with glibenclamide (20 μL) as the internal standard, and then 900 μL of acetonitrile was used to precipitate the protein. All samples were vortex–mixed for 5 min, then centrifuged at 15,000 *r*/min for 10 min. Then, 800 μL of supernatant was measured, blowed dry by nitrogen, and reconstituted with 100 μL of acetonitrile.

The Thermo Fisher Scientific Hypersil GOLD column (2.1 mm × 100 mm, 1.9 μm) was used as the analytical column; it was maintained at 30°C with 1.0 mmol/L of ammonium acetate (A) and mathanol (B) as the mobile phase, using glibenclamide as the internal standard (IS). The gradient elution program was operated as follows: 0–1.5 min, 55% B–65% B; 1.5–2.5 min, 65% B–80% B; 2.5–3.0 min, 80% B–90% B; 3.0–4.0 min, 90% B; 4.0–4.5 min, 90% B–55% B; 4.5–6.0 min, 55% B. The sample injection volume was 5.0 μL, and the flow rate set at 0.2 ml/min.

Mass spectrometry was operated with multiple reaction monitoring (MRM) in a positive ionization mode. The ion pair of POC is *m/z* 377.1 and 163.0; the declustering voltage (DP) is 28 eV; and the collision energy (CE) is 30 eV. The ion pair of glibenclamide is *m/z* 494.1 and 169.0, and the DP is 65 eV, the CE is 50 eV.

### Evaluation of Neurological Deficit

Neurological deficit was evaluated 24 h after reperfusion (blinded to experimenters). The scoring system was built on a 5–point scale system, described previously (Longa et al., 1989). The rating score was assessed by the following standard: no neurological deficit, 0; failure to extend left forepaw fully, one; spontaneous circling to the left, two; falling to the left, three; inability to walk spontaneously and coma of consciousness, 4. The higher score reflects a more severe brain injury. Neurological scores for each group were calculated using six rats according to a sample size calculation (α err prob = 0.05, 1–β err prob = 0.95) and references (Katayama et al., 2017; Lemarchand et al., 2019; Zheng et al., 2019).

### Measurement of Infarct Volume

Cerebral infarction volume was determined using TTC staining. After neurological status was examined, rats were euthanized under deep anesthesia with 20% ethyl carbamate. Brains tissues were rapidly isolated and sliced into 2 mm–thick sections. The brain slices were incubated for 20 min in 2% 2, 3, 5–triphenyl tetrazolium chloride (TTC) (Sigma, MO, The United States) at 37°C followed by fixation with 4% paraformaldehyde overnight. The TTC stained normal brain tissue deep red, whereas the infarcted area became pale white. The infarction and total brain volume were quantified using ImageJ software. The infarct volume was calculated as a ratio of the infarcted tissue relative to the total area of the whole–brain coronal section (Ye and Gao, 2008; Katayama et al., 2017). Six rats in each group were used according to a sample size calculation (α err prob = 0.05, 1–β err prob = 0.95) and references (Katayama et al., 2017; Lemarchand et al., 2019; Zheng et al., 2019).

### Hematoxylin and Eosin (HE) Staining

At 24 h after the treatment, rats from each group were anesthetized and perfused with 0.9% NaCl and 4% paraformaldehyde. Then, the brains were removed, collected, and post - fixed in 4% formaldehyde solution for 2 h, dehydrated, clarified and paraffin embedded. The sections were stained with HE after dewaxing. The histological examination was performed under an optical microscope; the nuclei in the sections were blue, while the cytoplasm was red.

### Oxidative Stress of Biomarker Assay

Assessment of oxidative stress is an important part of the pathology of cerebral ischemia-reperfusion injury. According to the manufacturer's instructions, the whole brain tissues (Kong et al., 2020) were homogenized, and then supernatants were obtained by centrifugation at 3,000 *r*/min for 20 min. The supernatants of brain tissues were used to measure the levels of superoxide dismutase (SOD) and malondialdehyde (MDA) by the SOD and MDA assay kit, respectively (Nanjing Jiancheng Bioengineering Institute). A microplate reader was used to measure the absorbance at 450 and 532 nm with a UV spectrophotometer. Six rats in each group were used after a sample size calculation (α err prob = 0.05, 1–β err prob within 0.80–0.95).

### ELISA Assay for IL–6, IL–1β, TNF–α, and TXA_2_

The whole brain tissues (Cai et al., 2018; Lemarchand et al., 2019) were homogenized with 0.01 M PBS solution (pH 7.4). Then, supernatants were obtained by centrifugation at 3,000 *r*/min for 20 min and collected for ELISA assay. In addition, the brain tissues and blood samples were collected for TXA_2_ test. According to the manufacturer's instructions, the levels of inflammatory cytokines (IL - 6, IL–1β, TNF–α; R&D Systems, Minneapolis, MN, United States) and TXA_2_ (Wuhan ColorfulGene Biological Technology Co., LTD) were detected using the commercial ELISA kits. The resultant colored products were measured the absorbance at 450 nm. Six rats in each group were used after a sample size calculation (α err prob = 0.05, 1–β err prob within 0.80–0.95) and references (Longa et al., 1989; Gai et al., 2019; Zheng et al., 2019).

### Statistical Analysis

Data were expressed as mean ± standard deviation (SD). One way analysis of variance (ANOVA) with *post - hoc* analysis was used to compare the mean values. For continuous data with uneven variance and a normal distribution–specifically, neurological scores and infarct volume data - the Friedman test was used. A probability value of *p* < 0.05 indicated statistically significant differences.

## Results and Discussion

### Synthesis and Characterization of POC

The design of a mutual prodrug has improved and even overcome the undesirable properties related to the drugs by changing the structure of drugs; the changes resulted in reduced side effects and increased activity. Now the next step of chemistry is the development of new drugs that exhibit a desired outcome, preferably in a rapid and straightforward approach (Tsogoeva, 2010). POC was synthesized by a two-step procedure. First, ozagrel was chlorinated into ozagrel chloride with thionyl chloride. The chlorination of ozagrel was conducted in an anhydrous environment to avoid generating impurities. Then, acyl chloride reacted with paeonol, together with a chemical heat release process, to form POC. Conjugation of paeonol to chlorinated ozagrel was conducted in an ice bath.

**FIGURE 3:**
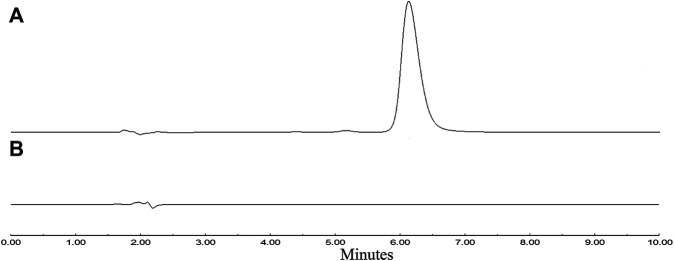
HPLC chromatogram. **(A)**: POC, **(B)**: blank solvent.

The synthesized compounds were confirmed by HR–MS, ^1^H–NMR, and ^13^C–NMR (Supplementary Figures S1–S3). The mass spectra studies on the targeted compound confirmed the parent peak and affirmed the molecular weight (376.15 g/mol). The NMR spectra data of the compound demonstrated characteristic chemical shifts of the ester bond, which predicted the POC synthesized successfully. The molecular formula of POC is C_22_H_20_N_2_O_4_, and the chemical structure of the conjugate POC was determined (Supplementary Figure S4). POC is easily soluble in methanol and ethanol, slightly soluble in ethyl acetate, and almost insoluble or insoluble in water; it has a melting point of 74–76°C and a maximum absorption wavelength of 280 nm. After purification and drying, a yield of 21.7% was achieved. The percentage purity was 98.5% by HPLC analysis (Figure 3).

### Determination of Partition Coefficient

The partition coefficient is an essential parameter in the development of drugs (Tsopelas et al., 2017). A drug’s partition coefficient is useful in estimating the distribution of drugs within the body and is emblematic of its ability to cross biological multiphase systems (Vraka et al., 2018). It has been successfully employed to model permeability, especially CNS and skin penetration (Young et al., 1988; Lipinski, 2004). To produce a pharmacological response in the brain, a drug molecule first has to cross a biological membrane, the BBB. It has been reported for many drugs have optimal passive brain entry *in vivo*, as indicated by their approximate partition coefficient of 2.0–3.5 (Waterhouse, 2003; Pike, 2009; Vraka et al., 2017). The result of this experiment showed that the partition coefficient of POC was 2.66, indicating its ability to enter the brain. However, to determine whether a compound can pass the BBB, both *in vitro* and *in vivo* assays need to be performed. A limitation of this study is that the BBB permeability was not assessed *in vivo*. Our research group is focusing attention now on brain–targeted preparations. We hope that POC can be systematically compared with brain–targeted preparations to explore the drug’s ability to pass through the BBB.

### PK Study

**FIGURE 4:**
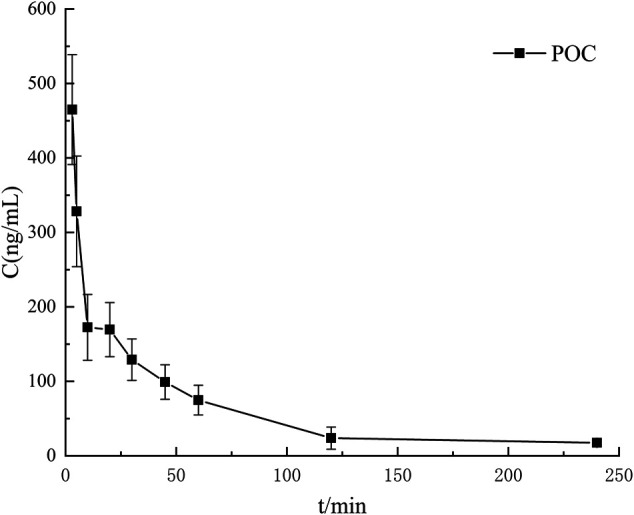
PK study. Concentration-time profiles after intravenous injection of POC solution.

The LC-MS/MS approach was successfully applied to *in vivo* pharmacokinetic study of POC after intravenous injection into rats at a dose of 4.5 mg/k. For analyte determination in plasma samples, a full method validation must include specificity (Supplementary Figure S5), linearity, recovery, matrix effect, precision, accuracy and stability in relation to the United States Food and Drug Administration (FDA) Bioanalytical Method Validation (Bioanalytical Method Validation Guidance for Industry, 2018). The results of method validation showed that a simple, accurate, and sensitive LC-MS/MS method was established for the quantification of POC and was successfully applied to plasma. The standard curve of POC was defined as *y* = 0.0008 *x* + 0.0037, *R* ^2^ = 0.9995, linear ranges 1–1,000 ng/ml. The mean plasma concentration - time curves are showed in Figure 4, and their estimated pharmacokinetic parameters are presented in Table 1.

**TABLE 1:** Pharmacokinetic parameters after intravenous injection of POC solution.

Parameters	POC solution
t_1/2_ (min)	53.46 ± 19.64
C_max_ (ng/ml)	464.92 ± 269.47
AUC_0-t_ (ng/mL⋅min^−1^)	15836.14 ± 3185.70
MRT_0-t_ (min)	51.89 ± 14.13
Cl (mL/min/kg)	276.75 ± 62.32

Pharmacokinetic results for the t_1/2_ (the time it takes for the concentration of the drug in plasma to drop by half) after intravenous injection were 53.46 ± 19.64 min, suggesting that POC eliminated quickly in body. C_max_ (peak concentrations) were 464.92 ± 269.47 ng/ml. The AUC_0-t_, MRT_0-t_, Cl were 15,836.14 ± 3,185.70 ng/mL/min, 51.89 ± 14.13 min, and 276.75 ± 62.32 ml/min/kg, respectively. However, the matabolism of POC should be investigated in more detail.

### Determination of Cell Viability

**FIGURE 5:**
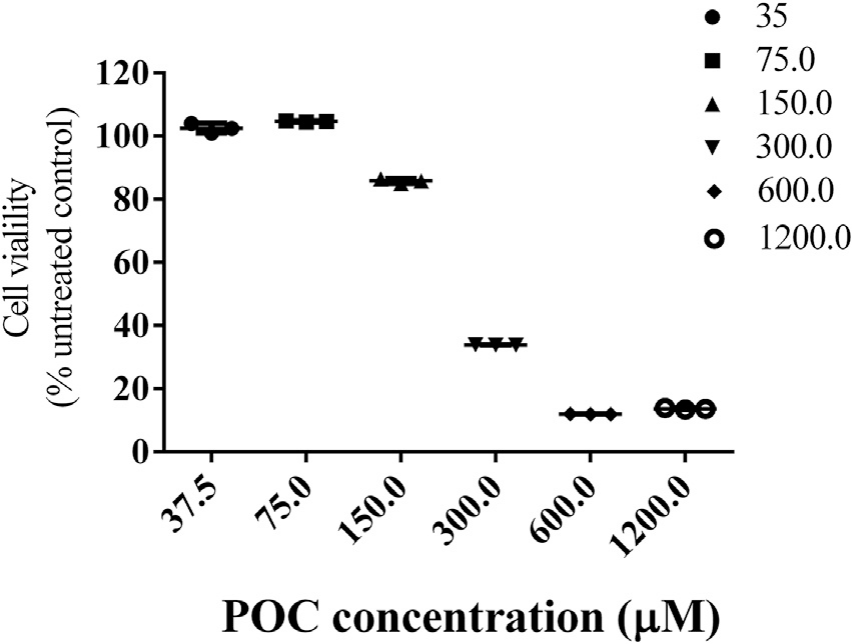
Cell viability. Effects of different concentrations of POC on the viability of PC12 cells. Data are expressed as means ± SD.

To detect the response of PC12 cells to POC itself, cells were exposed to varying concentrations, from 37.5 to 1,200.0 μM, of POC for 24 h. The obtained results shown in Figure 3 clearly demonstrate that increasing the concentration of the POC within a range decreased the viability of the treated PC12 cell, with a highly significant effect. Furthermore, POC resulted in no viability loss at a concentration of less than 75 μM in PC12 cells (Figure 5). POC had no toxic effect at a concentration of less than 75 μM for 24 h in PC12 cells.

### Evaluation of Neurological Deficit

**FIGURE 6:**
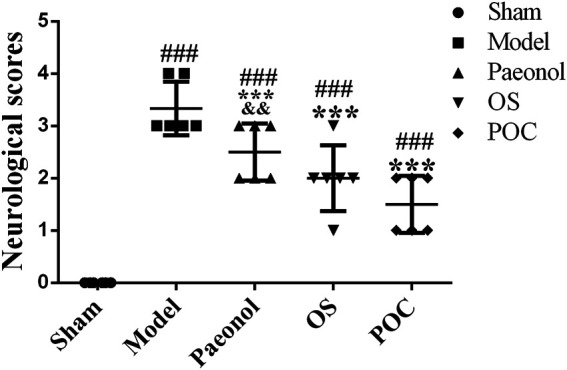
Neurological scores. On the *x* axis, the rats are divided into five groups: sham group, model group, paeonol group, OS group and POC group. The *y* axis displays the neurological scores at 24 h after reperfusion. Compared with the sham group, ^###^ *p* < 0.001. Compared with the model group, ^***^ *p* < 0.001. Compared with the POC group, ^&&^ *p* < 0.01 (n = 6).

In rats, changes of neurological function after ischemic stroke are reflected by neurobehavioral abnormality. After 24 h of cerebral ischemia - reperfusion injury, a neurological evaluation was performed according to the Longa method (0–four points) (Longa et al., 1989). The rats in the model, paeonol, OS and POC groups developed different severities of neurological deficits after undergoing MCAO. Those in the sham group had no neurobehavioural deficits and an average Longa method score of 0. However, the neurological deficits scores in the model, paeonol, OS, and POC groups were 3.33 ± 0.52, 2.50 ± 0.55, 2.00 ± 0.63, and 1.50 ± 0.55, respectively, (Figure 6). The neurological deficits of rats in the model group were significantly greater than the control group deficits. Compared with the model group, treatment with paeonol, OS and POC significantly reduced the neurological deficits (*p* < 0.001).

### Measurement of Infarct Volume

**FIGURE 7:**
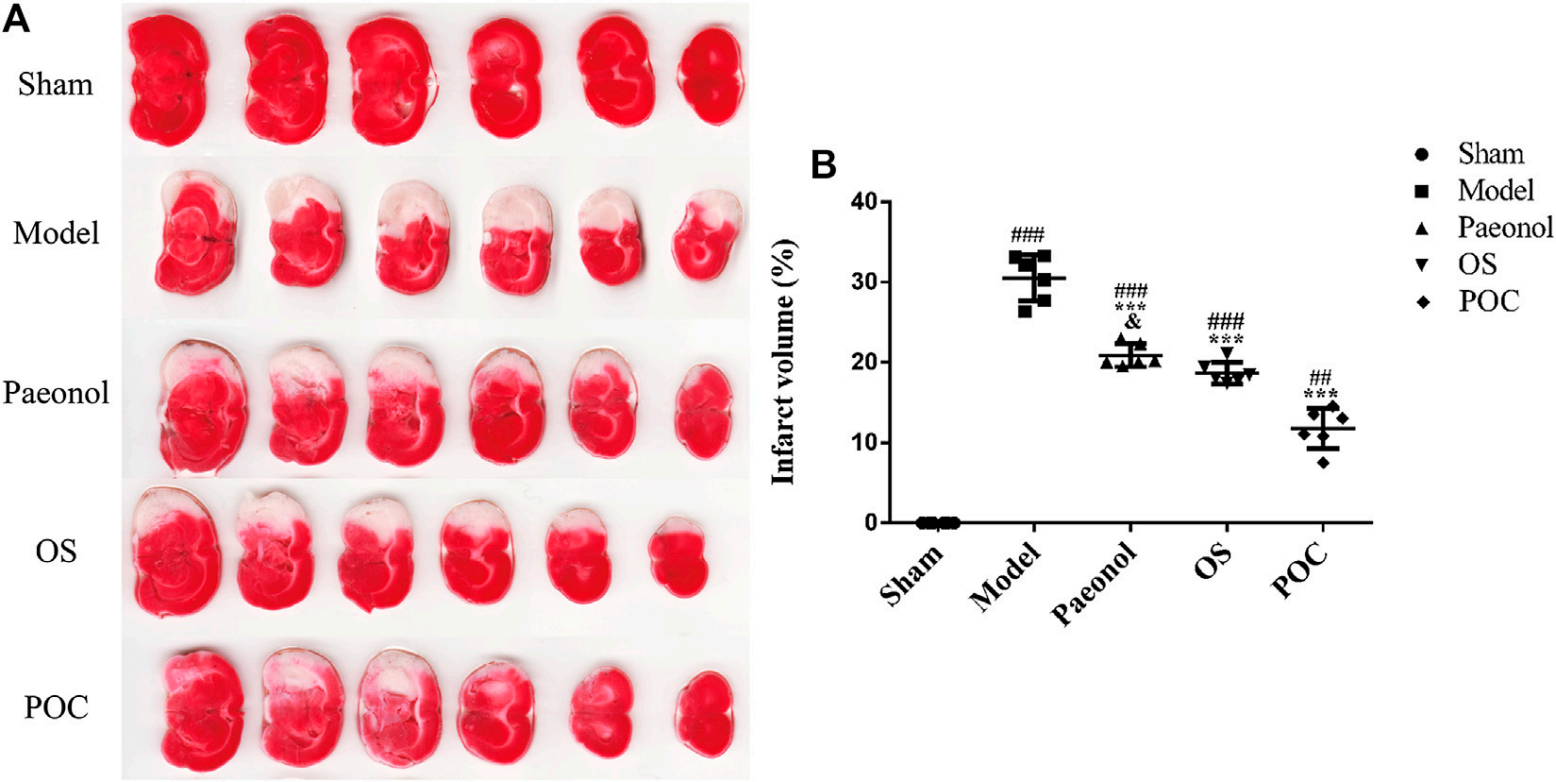
Cerebral infarction volume. **(A)** The TTC staining. Normal brain tissue was stained dark red, while the infarct area was stained pale white. **(B)** On the *x* axis, the rats are divided into five groups: sham group, model group, paeonol group, OS group and POC group. The *y* axis displays the cerebral infarction volume. Compared with the sham group, ^###^ *p* < 0.001, ^##^ *p* < 0.01. Compared with the model group, ^***^ *p* < 0.001. Compared with the POC group, ^&^ *p* < 0.05 (n = 6).

2, 3, 5–Triphenyltetrazolium chloride (TTC) staining is a convenient method for detecting cerebral infarction. TTC reacts with enzymes in the inner mitochondrial membrane and then is reduced by the enzymes into a red, lipid - soluble formazan. Therefore, viable tissue stains deep red while the infarct remains unstained (Joshi et al., 2004). In Figure 7, significant differences were observed in the cerebral infarct volume between rats from various experimental groups. The cerebral infarction volume percentages of rats in the model, paeonol, OS and POC groups were 30.49% ± 2.87%, 20.87% ± 1.43, 18.68% ± 1.35%, and 11.77% ± 2.53%, respectively. The cerebral infarction volume percentage in the model group was significantly greater than that of the sham group. Compared with the paeonol group, treatment with POC significantly reduced the cerebral infarction volume percentage (*p* < 0.05).

The water content in each brain hemisphere should be determined as a measure of cerebral edema. The brain tissues should be weighed before (wet weight) and after (dry weight) drying in 80°C for 72 h to a constant weight. Water content in brain is then calculated as (wet weight - dry weight)/dry weight × 100% (Todd et al., 2006; Li et al., 2009). One limitation of this study is that we did not correct for the effect of brain edema/swelling.

### Histopathologic Changes in Brain Cortex

**FIGURE 8:**
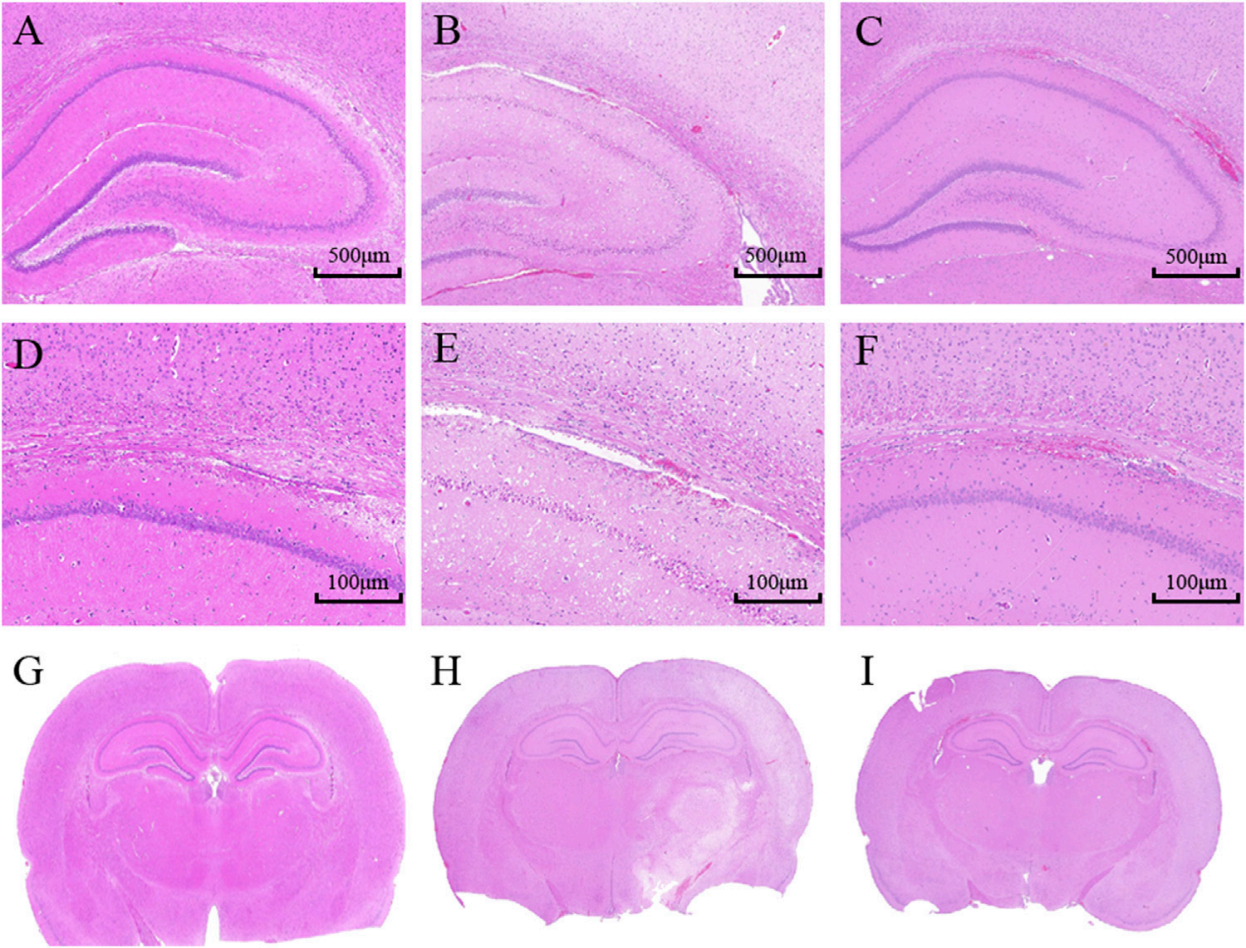
Histopathological assessment in the cortex of rats; **(A– C)** HE 10×; **(D–F)** HE 2.0×; **(G–I)** HE whole brain. **(A, D, G)** Sham group; **(B, E, H)** Model group (24 h); **(C, F, I)** POC group (n = 3 in each group).

HE 10× showed that the structure in the cerebral cortex was complete in the sham group. Neuronal cells were uniformly distributed and neatly arranged. No visible pathological changes in the brain were observed. However, in model group, the cerebral infarct region emerged with extensive degeneration, including neuronal cells loss, nuclei condensation with deep coloration, and the formation of vacuoles. The POC group had only marginal neuronal cells damage compared with the model group. Neuronal cells were well arranged and clearly outlined. A small number of cells displayed nuclei condensation and deep coloration. HE 2.0× and HE whole brain in model rats both showed a white cerebral infarction area on the right side of the whole brain slice, which confirmed POC has a significant therapeutic effect (Figure 8).

### Oxidative Stress

Oxidative stress is a pivotal cellular response to cerebral ischemia - reperfusion (Zhao et al., 2014). The increased expression of SOD catalyzes the dismutation of the superoxide (O_2_^−^) radical into either ordinary molecular oxygen (O_2_) or hydrogen peroxide (H_2_O_2_), which inhibites oxygen free radicals production. In addition, MDA is a marker for high levels of oxidative stress (Pan et al., 2017).

**FIGURE 9:**
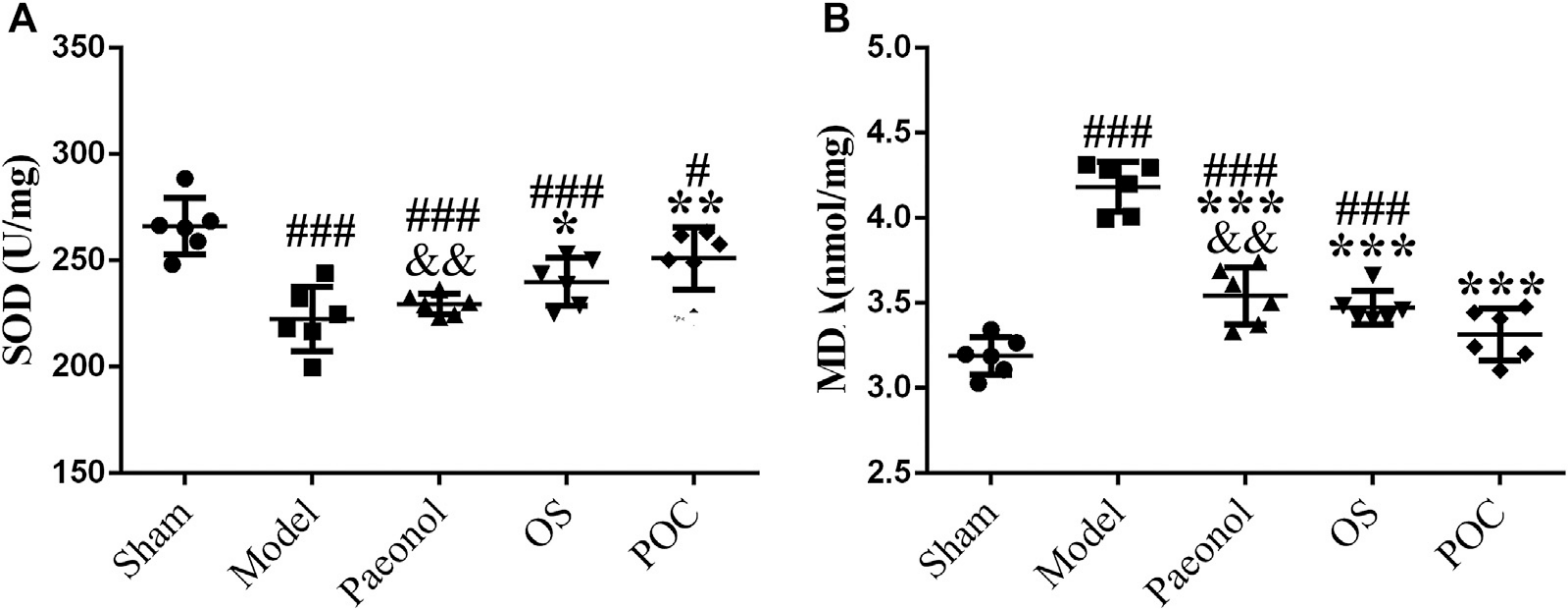
Oxidative stress. On the *x* axis, the rats are divided into five groups: sham group, model group, paeonol group, OS group, and POC group. The *y* axis displays **(A)** SOD (U/mg) and **(B)** MDA (nmol/mg). Compared with the Sham group, ^###^ *p* < 0.001, ^#^ *p* < 0.05. Compared with the Model group, *** *p* < 0.001, ** *p* < 0.01, * *p* < 0.05. Compared with the POC group, ^&&^ *p* < 0.01 (n = 6).

Compared with rats in the sham group, the levels of oxidative stress were remarkably increased in rats in the model group; this was reflected mainly through lower SOD levels (Figure 9A, 1 × 5 groups ANOVA *F* (4, 25) = 11.711, *p* < 0.001) and higher MDA levels (Figure 9B, 1 × 5 groups ANOVA *F* (4, 25) = 47.219, *p* < 0.001), indicating that the oxidative products increased after MCAO, while the activity of the main active enzymes that scavenged free radicals decreased, and the antioxidant capacity decreased. For drug groups, rats in the POC group showed the highest SOD levels and lowest MDA levels compared with the paeonol and OS groups. Besides, results for the POC group differed significantly compared with the paeonol group (*p* < 0.01) but not the sham group (*p* > 0.05). All these results confirmed that POC has strong anti–oxidative stress ability in cerebral ischemia reperfusion injury, which can be a protective agent to reduce oxidative stress induced by ischemic stroke.

### ELISA Assay Results

In the acute stage of cerebral ischemic, the damage of the inflammatory reaction to brain tissue may be more serious than that of ischemic itself. The inflammatory response is initiated by leukocyte infiltration and activation of microglia, astrocytes and endothelial cells after ischemic stroke (Zaleska et al., 2009). Because of the inflammatory cytokines IL–1β, IL–6, and TNF–α play significant roles in damging brain tissue after stroke, reducing the levels of these inflammatory cytokines may hold promise as a strategy for improveing stroke outcomes (Liu et al., 2016; Qiu et al., 2016; Zhou et al., 2017). TXA_2_ has strong effects on vascular contraction and vascular endothelial cell dysfunction, which promote ischemic stroke injury. Therefore, reducing the production of TXA_2_ may ameliorate neural injury in ischemic stroke (Zhou et al., 2014; Fei et al., 2017).

**FIGURE 10:**
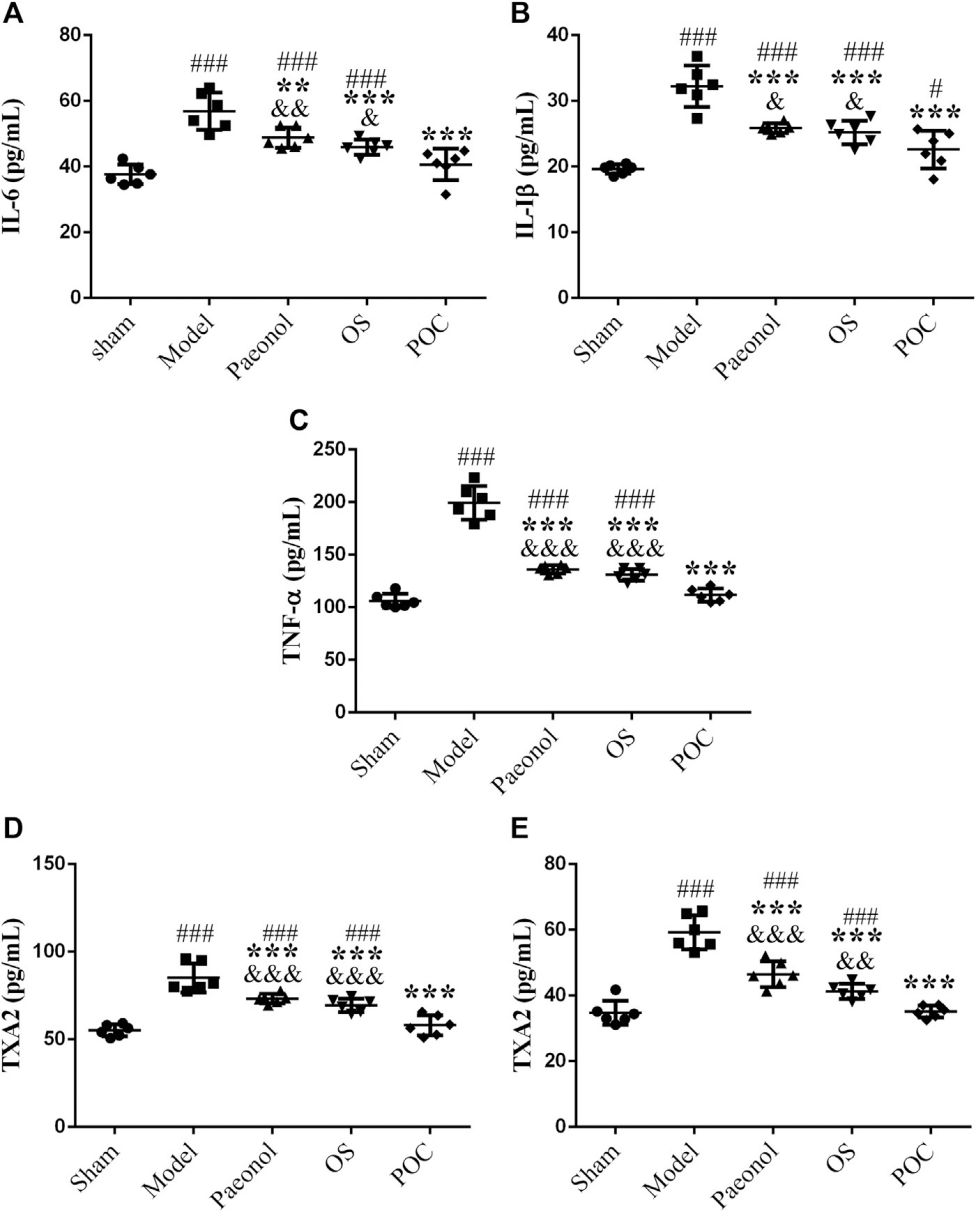
The levels of inflammatory cytokines and TXA_2._ On the *x* axis, the rats are divided into five groups: sham group, model group, paeonol group, OS group and POC group. The *y* axis displays **(A)** IL–6 (pg/ml), **(B)** IL–1β (pg/ml), **(C)** TNF–α (pg/ml), **(D)** TXA_2_ (pg/ml) of brain tissue, **(E)** TXA_2_ (pg/ml) of plasma. Compared with the sham group, ^###^ *p* < 0.001, ^#^ *p* < 0.05. Compared with the model group, *** *p* < 0.001. Compared with the POC group, ^&&&^ *p* < 0.001. (n = 6).

To identify an inflammatory reation after cerebral ischemic, the levels of IL–1β, IL–6, TNF–α and TXA_2_ were examined by ELISA. In model group, cerebral ischemia induced a remarkable inflammatory response compared with the sham rats, specifically reflected in the higher IL–six levels (Figure 10 A, 1 × 5 groups ANOVA *F* (4, 25) = 21.253, *p* < 0.001), higher IL - 1β levels (Figure 10 B, 1 × 5 groups ANOVA *F* (4, 25) = 29.204, *p* < 0.001), and higher TNF - α levels (Figure 10 C, 1 × 5 groups ANOVA *F* (4, 25) = 107.261, *p* < 0.001). IL–1β is considered to be a neurotoxic mediator, which induces neuronal apoptosis (Shichita et al., 2012). Reports showed that the inhibition of IL - 1β production reduces infarction volume (Yamasaki et al., 1995; Abulafia et al., 2009). TNF–α excutes its neurotoxic function by promoting the production of oxygen free radicals and excitatory amino acids, which ultimately results in neuronal cell death. Since neutralizing anti–TNF antibodies can also suppress the downstream production of IL–6, it is plausible that the protective effect of inhibiting TNF occur in part by inhibiting the production of other downstream cytokines (Meistrell et al., 1997). Inflammatory cytokines influence each other, which may cause the expansion of local inflammation and aggravate brain tissue damage. As shown in scatter plots, the three different drug groups decreased the release of inflammatory cytokines (*p* < 0.001). The POC group showed statistical differences compared with the other two drug groups (*p* < 0.05). Therefore, POC can inhibit the expression of three markers of inflammatory cytokines, thereby reducing the inflammatory reation, effectively reducing cerebral ischemic damage to play a protective role in. In brain (Figure 10 D, 1 × 5 groups ANOVA *F* (4, 25) = 32.480, *p* < 0.001) and the blood (Figure 10 E, 1 × 5 groups ANOVA *F* (4, 25) = 46.940, *p* < 0.001), the three different drugs increased the levels of TXA_2_. POC can inhibit platelet adhesion, aggregation, and play an anti–thrombotic effect by inhibiting the synthesis of TXA_2_. This results demonstrated that two monomer compounds, paeonol and OS, can improve ischemic stroke results and reduce neurological deficits. However, only the POC group showed no significantly different from sham group (*p* > 0.05). Our results suggest that POC has the potential to be developed into anti–ischemic stroke agent.

## Conclusion

In conclusion, POC, a novel therapeutic drug for ischemic stroke, was successfully synthesized by conjugating of paeonol and ozagrel. The study *in vivo* showed a visible improvement of the neurological score and infarct volume in POC–treated rats after MCAO. Furthermore, the neuroprotective effects of POC were through increasing number of survival cells especially neurons, altering levels of oxidative stress markers and inflammatory cytokines in ischemic brain as well as decreasing levels of vasoconstrictor TXA_2_ in brain lysates and plasma. Overall, all these results suggest POC may become a new candidate drug for cerebral ischemic stroke treatment. In the future, we will research the mechanism used by POC to protect against cerebral ischemia–reperfusion injury and experiments with different preparations, such as brain–targeted prepartion, to provide more evidence for its use in cerebrovascular disease.

Weighed an appropriate amount of POC in methanol solution, and diluted the solution configured to 100 ng/ml for mass spectrometry analysis. The molecular formula of POC is C_22_H_20_N_2_O_4_. The molecular weight is 376.14 by calculating. Known from the MS1 figure [M + H]^+^ m/z = 377.1486, which predicted the POC synthesized successfully. MS2 indicated the possible cleavage of POC compound.

^13^C-NMR (100MHz, DMSO-*d*_*6*_) *δ*: 195.4 (C-7), 164.6 (C-13′), 163.3 (C-4), 150.8 (C-2), 145.9 (C-11′), 140.6 (C-1′), 137.5 (C-10′), 133.3 (C-4′), 132.4 (C-1), 129.0 (C-3′, C-5′), 128.8 (C-9′), 127.9 (C-2′,C-6′), 123.2 (C-8′), 119.8 (C-12′), 117.4 (C-6), 111.8 (C-5), 109.4 (C-3), 55.9 (C-8), 49.1 (C-7′), and 29.1 (-OCH_3_).

^1^H-NMR (400 MHz, DMSO-*d*_*6*_) *δ*: 7.92 (1H, d, H-6), 7.85–7.79 (4H, H-2′, 6′, 9′, 11′), 7.32 (2H, H-3′, 5′), 7.22(1H, s, H-10′), 6.98 (1H, dd, H-5), 6.94 (1H, H-3), 6.91–6.87 (2H, H-8′, 12′), 5.26 (2H, s, H-7′), 3.85 (3H, s, H-5), and 2.48 (3H, s, H-8).

## Data Availability Statement

The original contributions presented in the study are included in the article/Supplementary Material, further inquiries can be directed to the corresponding author.

## Ethics Statement

Male Sprague Dawley (SD) rats weighing among 220 g with 230 g (Anhui Medicine University, Hefei, China, certificate number: SCXK20190003) were housed under ad libitum access to food and water with a standardized light-dark cycle condition (25 ± 2°C, 12 h light/dark cycle). All of the experimental protocols were performed in accordance with the Institutional Animal Care and Use Committee of Anhui University of Chinese Medicine (Animal ethics number: AHUCM–rats–2019001).

## Author Contributions

JZ and MJ performed the majority of the experiment and analyzed the data. HZ and LH drafted and revised the manuscript. YJ, WC, JW, and ZZ supported several experiments. CP, LH, and WC surpervised the research and revised the manuscript.

## Conflict of Interest

 Author YJ was employed by Chaohu Jinchen Pharmacy Co., Ltd., Shanghai Haihong Industrial Group (Chaohu 23800, China).

The remaining authors declare that the research was conducted in the absence of any commercial or financial relationships that could be construed as a potential conflict of interest.
