# Supplementary figures and images for: Synthesis of Paeonol-Ozagrel Conjugate: Structure Characterization and In Vivo Anti-Ischemic Stroke potential
Source: Front Pharmacol. 2021 Feb 1;11:608221. doi: 10.3389/fphar.2020.608221 (PMC7883289; doi:10.3389/fphar.2020.608221)

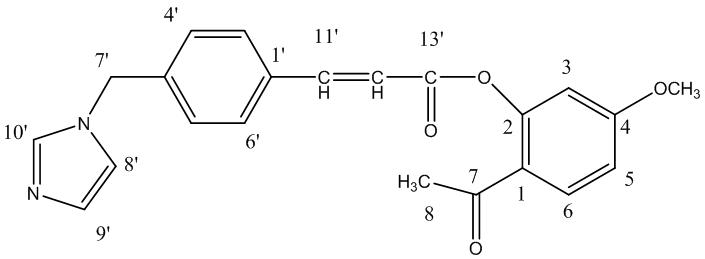

Supplement: Supplementary file 1 [file image1.tif]

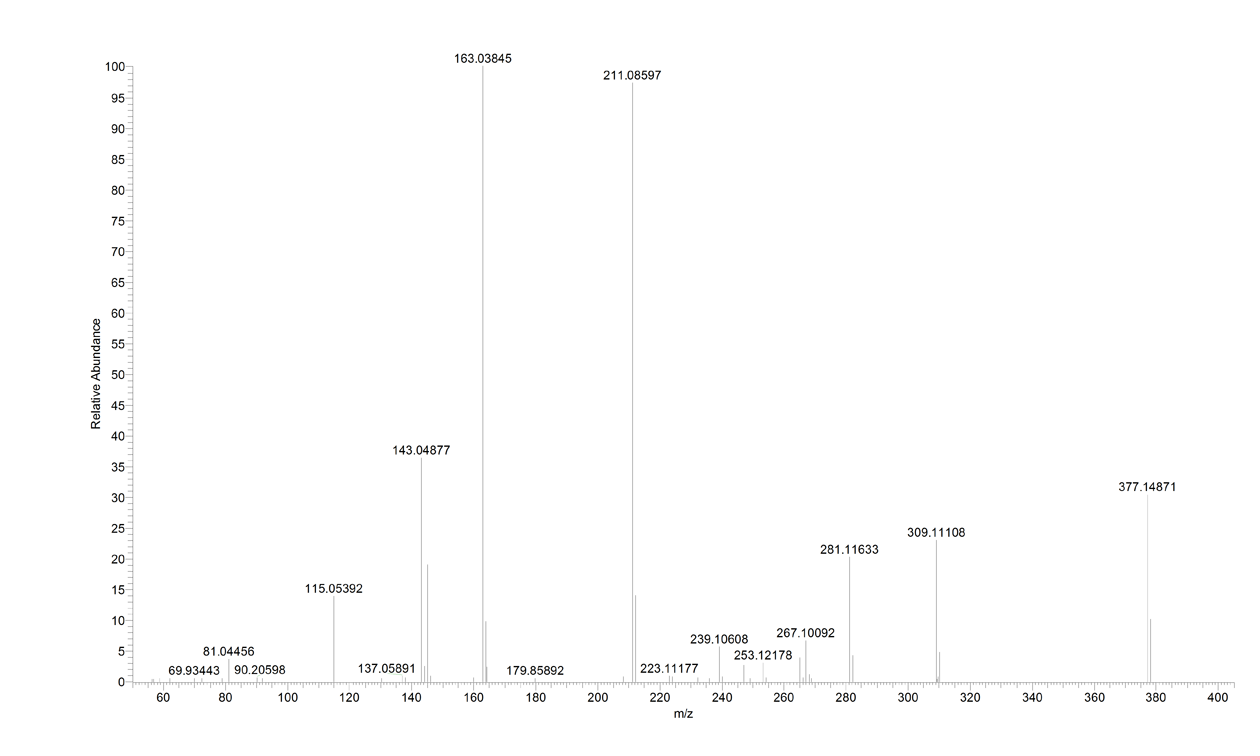

Supplement: Supplementary file 2 [file image2.tif]

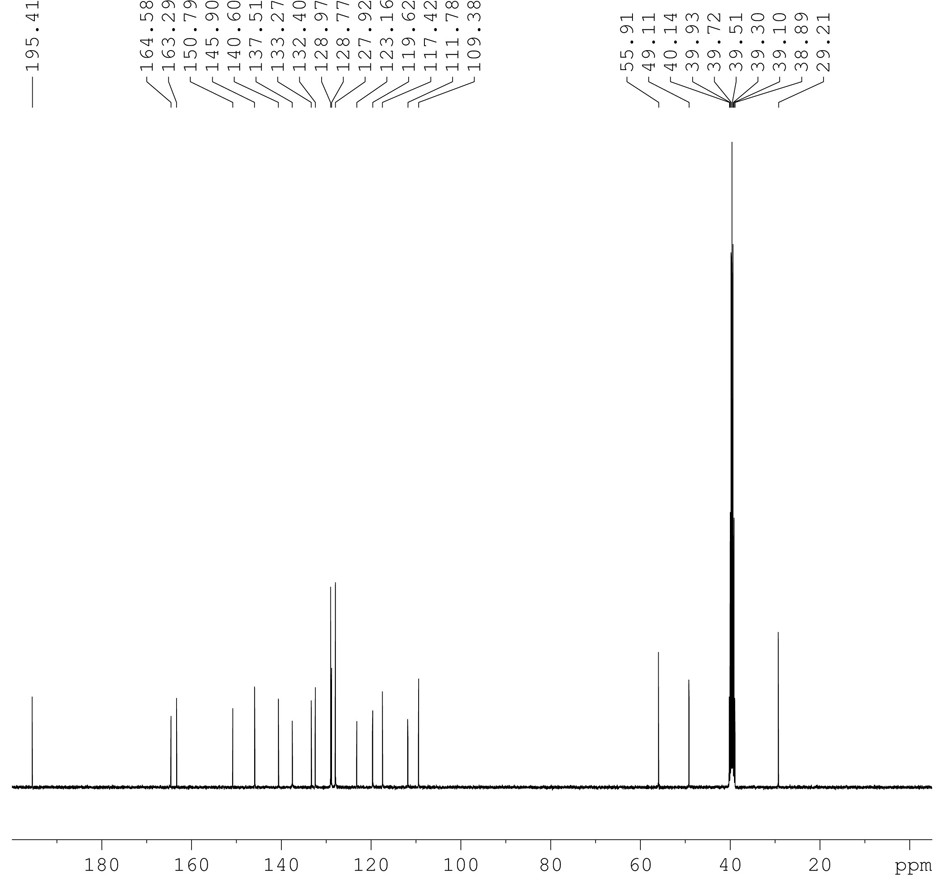

Supplement: Supplementary file 3 [file image3.tif]

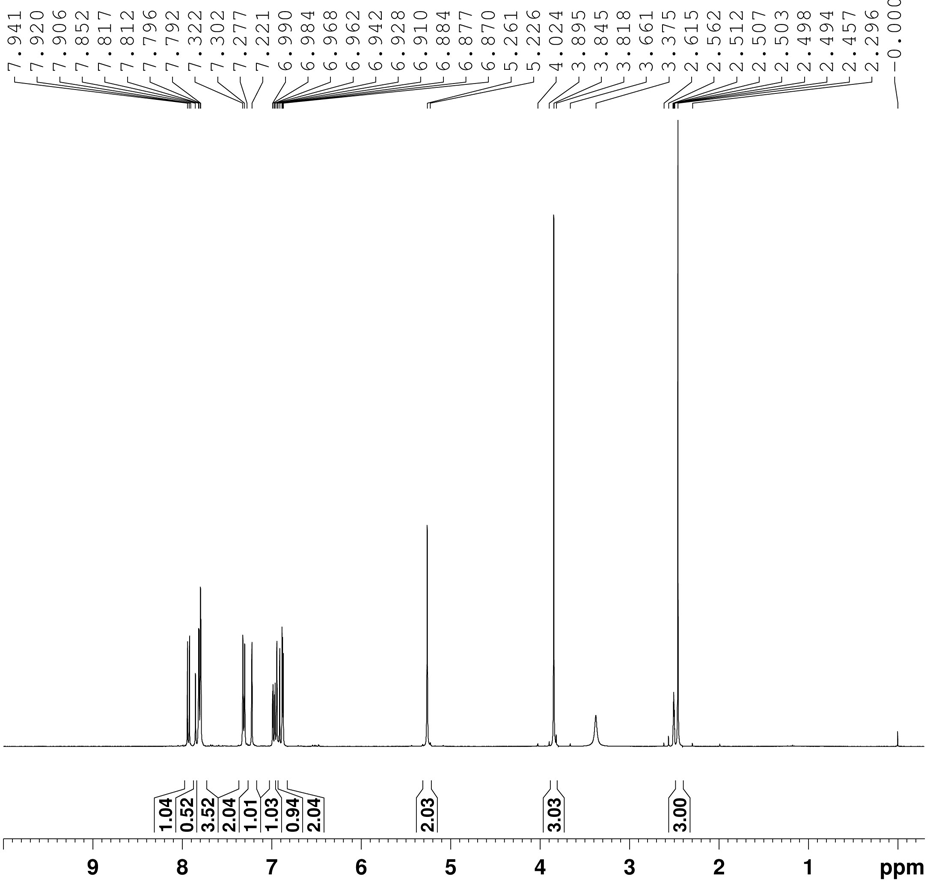

Supplement: Supplementary file 4 [file image4.tif]

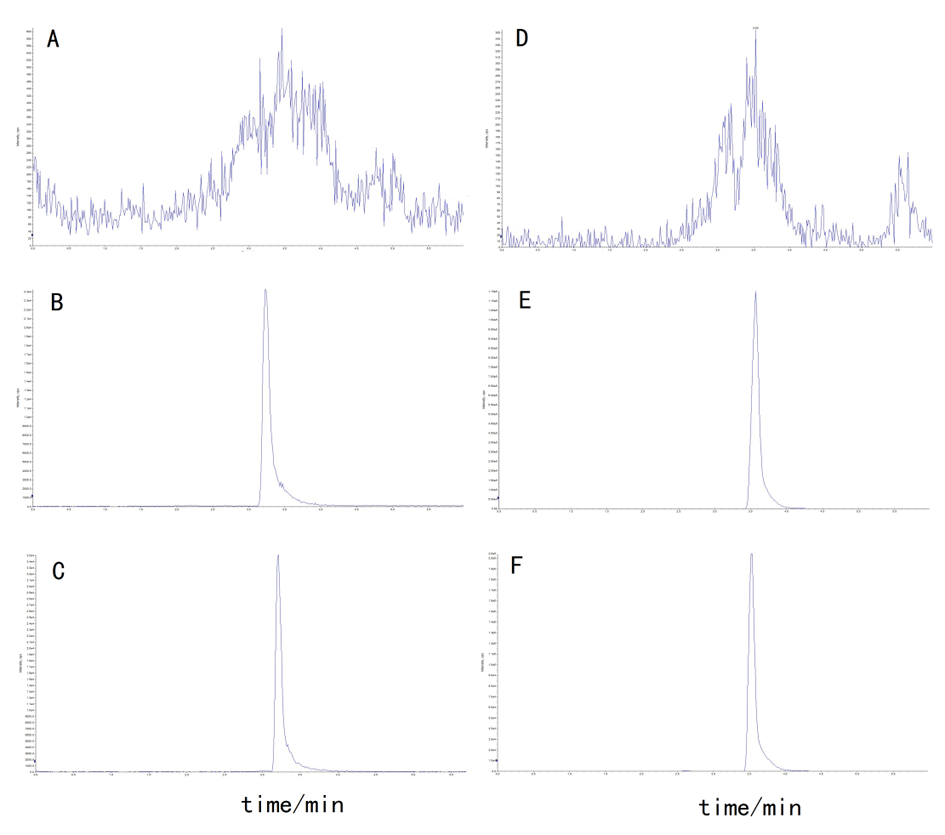

Supplement: Supplementary file 5 [file image5.tif]
